# Alpha-2 Adrenergic Agonists Reduce Heavy Alcohol Drinking and Improve Cognitive Performance in Mice

**DOI:** 10.1523/ENEURO.0368-25.2026

**Published:** 2026-01-29

**Authors:** Sema G. Quadir, Lauren Lepeak, Sophia Miracle, Roberto Collu, Olivia Velte, Yingchu He, Zeynep Ozturk, Christian D. Rohl, Valentina Sabino, Pietro Cottone

**Affiliations:** Laboratory of Addictive Disorders, Department of Pharmacology, Physiology and Biochemistry, Department of Psychiatry, Boston University Chobanian & Avedisian School of Medicine, Boston, Massachusetts 02118

**Keywords:** addiction, ADHD, ethanol, guanfacine, withdrawal

## Abstract

Alcohol use disorder (AUD) is one of the top behavioral causes of global disease burden in the United States. Repeated cycles of alcohol intoxication and abstinence induce neuroplastic alterations which induce excessive drinking and cognitive impairments. A system deeply dysregulated by chronic drinking is norepinephrine (NE). At moderate levels, NE has beneficial effects on cognition and behavior, mediated by the α2 adrenergic receptor (AR) subtype. Whether α2 AR activation blunts alcohol consumption in models of heavy drinking has not been determined, and whether α2 AR activation improves cognitive performance following chronic alcohol consumption is unknown. Here, we show that the α2 AR agonist clonidine worsens ethanol-induced hypothermia and sedation in male mice, while the more selective α2 AR agonist guanfacine is devoid of these effects. We also observed that, in male and female mice, while both clonidine and guanfacine reduce heavy alcohol drinking, guanfacine does so with higher potency. Furthermore, guanfacine improved cognitive performance in a temporal order test and, partially, in a novel object recognition test but had no effect in a novel spatial location test, in male and female ethanol-experienced mice. Finally, we found that chronic intermittent ethanol drinking increases the number of persistently activated NE neurons in both the locus ceruleus and the nucleus of the tractus solitarius, in both male and female mice. Our results highlight a central role for the α2 AR system in heavy alcohol drinking and associated cognitive deficits, suggesting that α2 AR stimulation may represent a viable pharmacological strategy to treat AUD.

## Significance Statement

Our data show a major role for the norepinephrine system and the α2 adrenergic receptor subtype in regulating ethanol consumption and improving cognition and provide support for the use of guanfacine in the management of AUD.

## Introduction

Alcohol use disorder (AUD) affects 29 million individuals and causes over 140,000 deaths annually in the United States (NSDUH, 2021; CDC, 2022). Patients with AUD show compulsive drinking as well as profound cognitive impairments, including deficits in episodic memory, attention, and cognitive flexibility, all of which can hinder recovery. Currently only three modestly effective pharmacological therapies are available for AUD (Reus et al., 2018; Koob, 2021; Mason and Heyser, 2021); hence, new treatments based on pathophysiological changes occurring following chronic alcohol consumption are warranted.

Norepinephrine (NE), a major neurotransmitter of the central and peripheral nervous system, plays an essential role in the regulation of arousal, attention, cognitive function, and stress reactions (Berridge and Waterhouse, 2003; Aston-Jones and Cohen, 2005; Rinaman, 2011; Sara and Bouret, 2012). NE neuronal cell bodies are confined to a few relatively small brain areas. The two primary NE ascending projections are the dorsal noradrenergic bundle (DNB), originating from the A6 locus ceruleus (LC) nucleus, and the ventral noradrenergic bundle (VNB), originating mainly from the A2 nucleus of the tractus solitarius (NTS; Moore and Bloom, 1979; Robertson et al., 2013). These nuclei send widespread projections to various brain regions (Moore and Bloom, 1979). NE exerts its central effects according to an inverted U-shaped curve: whereas moderate NE levels generally have beneficial effects on behavior and cognition, both insufficient and excessive NE levels translate into impaired functions (Arnsten and Li, 2005; Arnsten, 2006). While the detrimental effects of high NE levels are mediated by the low affinity α1 adrenergic receptor (AR; Arnsten and Goldman-Rakic, 1985a,b; Cai et al., 1993; Birnbaum et al., 1999; Ramos et al., 2005), the effects of moderate NE levels mostly occur via the activation of the high affinity α2 AR subtype (Wang et al., 2007; Harris et al., 2018).

Chronic alcohol consumption induces profound neuroadaptations in the NE system in corticolimbic areas, which in turn cause allostatic changes that trigger and sustain excessive drinking (Koob, 2003, 2008, 2009, 2015; Walker et al., 2008; Breese et al., 2011). Repeated alcohol intoxication followed by withdrawal increases central levels of NE and its metabolites both in individuals with AUD and in the rodent brain (Karoum et al., 1976; Hawley et al., 1981; Patkar et al., 2003; Rasmussen et al., 2006; Jaime and Gonzales, 2019), hence the increasing interest in the modulation of NE systems as a potential target for excessive drinking (Haass-Koffler et al., 2018; Varodayan et al., 2025). *α1 AR antagonism* (e.g., with prazosin) has been shown to reduce alcohol drinking, craving, and stress-induced reinstatement of alcohol seeking in rodents and to facilitate abstinence in humans (Walker et al., 2008; Rasmussen et al., 2009; Gilpin and Koob, 2010; Le et al., 2011; Verplaetse et al., 2012; Fox et al., 2012b; Froehlich et al., 2015; Simpson et al., 2018). Much less focus has instead gone into the investigation of α2 ARs; *α2 AR agonism* (e.g., with clonidine) was shown to block stress-induced reinstatement of nicotine, alcohol, heroin, and cocaine seeking, while the α2 AR antagonist yohimbine is known to trigger reinstatement (Opitz, 1990; Erb et al., 2000; Lee et al., 2004; Le et al., 2005; Zislis et al., 2007; Yamada and Bruijnzeel, 2011; Rasmussen et al., 2014). A recent clinical trial evaluating the effects of the α2 AR agonist guanfacine in women with AUD has shown promising effects (https://clinicaltrials.gov/study/NCT03137082?tab=results). Whether α2 AR activation blunts alcohol consumption in mouse models of heavy drinking has not been determined.

The imidazoline derivative α2 AR agonist clonidine is an older antihypertensive drug well known for its efficacy in treating withdrawal symptoms from opiate and alcohol, thanks to its ability to alleviate autonomic symptoms (Gold et al., 1978; Washton and Resnick, 1980; Parale and Kulkarni, 1986; Gossop, 1988; Opitz, 1990); however, common side effects include sedation and hypotension (Kumar et al., 2014). Guanfacine, a more selective α2 AR agonist, has a 20 year safety record in adults (Sorkin and Heel, 1986); guanfacine is used to treat attention deficit hyperactivity disorder (ADHD) and tics in children due to its cognitive-enhancing effects and its ability to improve prefrontal cortex (PFC) function (Arnsten et al., 1988; Hunt et al., 1995; Horrigan and Barnhill, 1996; Scahill et al., 2001; Boon-yasidhi et al., 2005; Connor et al., 2014; Arnsten, 2020). Guanfacine has been shown to reduce craving for cocaine and nicotine both in humans and in animals (Weinshenker and Schroeder, 2007; Fox et al., 2012a; Perez et al., 2020). While the efficacy and the mechanism of α2 AR agonists in blunting physical symptoms of withdrawal are well understood (Trzaskowska et al., 1986; Riihioja et al., 1997), their potential effects on heavy alcohol drinking and associated cognitive performance have instead not been sufficiently investigated (Opitz, 1990; Rasmussen et al., 2014), despite the high translational potential.

In this study, we compared the effects of guanfacine to those of the older agonist clonidine first on ethanol-induced hypothermia and sedation, then on ethanol intake using a mouse model of chronic, intermittent heavy alcohol drinking. Additionally, we investigated the effects of guanfacine on cognitive performance in ethanol drinking mice and began looking at the neuroadaptations in noradrenergic brain regions induced by chronic, intermittent heavy ethanol drinking. We hypothesize that the activation of α2 ARs will selectively reduce alcohol drinking and that guanfacine will do so at lower doses. We also hypothesize that guanfacine will have a beneficial effect on cognitive tasks that depend on the PFC in ethanol-experienced animals.

## Materials and Methods

### Subjects

Male and female C57Bl/6J mice (7–8 weeks old upon arrival) were purchased from The Jackson Laboratory. Mice were single-housed with Teklad Diet 2918 and water *ad libitum* in a humidity- and temperature-controlled AAALAC-approved vivarium on a 12 h reverse light/dark cycle (lights off at 10:00 A.M.). Male and female mice were housed in the same vivarium room, though at a distance from each other. Animal numbers are specified in each experimental section below. All experiments were conducted during the animals’ dark cycle. Because animals were housed individually, which may influence stress-related physiology and behavior, potential effects of single housing on the measures reported here should be considered when interpreting the results. Procedures adhered to the National Institutes of Health *Guide for the Care and Use of Laboratory Animals* and *the Principles of Laboratory Animal Care* and were approved by the institutional Animal Care and Use Committee.

### Drugs

The ethanol solution for injections (20% w/v, administered i.p.) was prepared diluting 200-proof ethanol (Pharmco-Aaper) in isotonic saline. The ethanol solution for drinking experiments (20% v/v) was prepared using 200-proof ethanol (Thermo Fisher Scientific) and tap water. Then, 1.15% (w/v) sucrose (Sigma-Aldrich) was diluted in tap water. Clonidine hydrochloride was purchased from Sigma-Aldrich and dissolved in physiological saline; guanfacine hydrochloride was from TCI America; concentrated stock aliquots were made in 5% dimethylsulfoxide (DMSO, Sigma-Aldrich) in water; aliquots were then diluted to the final concentrations using physiological saline, and the final concentration of DMSO was negligible. Clonidine and guanfacine were administered intraperitoneally in a volume of 10 ml/kg of body weight.

### Temperature measurements

Mice were habituated to the testing room for 1 h prior to the beginning of the test and they were housed individually during the experiment. Body temperature was monitored using an Acorn Temp TC Thermocouple Meter fitted with a RET-3 rectal probe for mice (Kent Scientific), which was inserted into the animals’ rectum to a depth of ∼2 cm. An initial temperature measurement was made just prior to the injection of clonidine (0, 20, 80, 160 µg/kg, i.p.) or guanfacine (0, 12.5, 25, 50 µg/kg, i.p. 30 min later, ethanol (3.6 g/kg, i.p.) or an equivalent volume of saline were administered, in a between-subjects design. Since this dose of ethanol results in loss of righting response (see below), ethanol-treated mice were placed on their backs, while saline-treated animals were left free in the cages. Temperature was then recorded 30, 60, 90, 120, and 240 min after ethanol administration. The tests with saline and with ethanol were run on two different days and on different animals. The total number of mice was as follows: 26 mice (6–7/group) for clonidine/saline, 28 mice (6–8/group) for clonidine/ethanol, and 27 mice (6–7/group) for both guanfacine/saline and guanfacine/ethanol.

### Loss of righting reflex

Ethanol-induced loss of righting reflex (LORR) is a measure of the sedative effect of ethanol, and it was determined as previously described (Crabbe et al., 2006; Linsenbardt et al., 2009; Valenza et al., 2016). The same ethanol-treated mice from the above experiment were used for the LORR experiment. Thirty minutes after clonidine or guanfacine administration, mice were injected with ethanol (3.6 g/kg, i.p.) and placed on a V-shaped surface (the food hopper portion of the cage wire tops). Latency to lose the righting reflex and sleep duration were recorded using a stopwatch. The latency was defined as the time between the ethanol injection and the time when the mouse was unable to right itself from a supine position for at least 30 s. Mice remained undisturbed until they could right themselves onto all four paws twice within a 30 s period. Mice that did not fall asleep following ethanol injection were excluded (one in the clonidine experiment, three in the guanfacine experiment). The total number of mice was 30 (7–8/group) for clonidine and 22 (4–7/group) for guanfacine.

### Mouse intermittent access to two bottle choice (IA2BC)

Upon arrival, mice were habituated to drink water out of Corning falcon 50 ml conical-bottom centrifuge tubes (Fisher Scientific) equipped with #6R rubber stoppers with 2.5″ long straight metal double ball bearing sipper tubes (Ancare; “bottles”). Mice were then given intermittent access using a two bottle choice (IA2BC) paradigm, during which time one water bottle was replaced with a bottle containing 20% (v/v) ethanol on alternating days for 24 h, as done previously (Hwa et al., 2011; Navarro et al., 2019; Quadir et al., 2020; Lepeak et al., 2023). Briefly, at the beginning of the dark cycle, preweighed bottles (one ethanol, one water) were provided, and 24 h later bottles were removed and weighed to calculate intake. Additional cages and sets of bottles were used to ensure negligible spillage during cage handling. Controls were given water only. Drug testing on ethanol drinking began after a minimum of 12 weeks of IA2BC. On drug injection days, bottles and food were removed at injection time and returned after pretreatment time (30 min). Alcohol and water weights were recorded at 2, 6, and 24 h, common time points in the literature (Sabino et al., 2013; Newman et al., 2018; Quadir et al., 2021a,b; Lepeak et al., 2023). Seventeen mice (8 males, 9 females) were used in the clonidine ethanol drinking experiment. Twenty mice (9 males, 11 females) were used in the guanfacine ethanol drinking experiment. Thirty-two mice (16 males, 16 females, *n* = 7–9/group) were used in the LC immunohistochemistry (IHC) experiment; 30 mice (13 males, 17 females, *n* = 4–9/group) were used in the NTS IHC experiment.

In separate cohorts of (ethanol-naive) mice, the above IA2BC procedure was performed in an identical way, except that the bottles contained 1.15% (w/v) sucrose instead of ethanol, for at least 7 weeks. Nineteen mice (9 males, 10 females) were used for the clonidine sucrose drinking experiment. Nineteen mice (8 males, 11 females) were used for the guanfacine sucrose drinking experiment.

### Cognitive tests

Guanfacine was tested for its ability to influence cognitive performance in ethanol-experienced mice, using tests of object recognition memory that required judgments about either recency, object identity, or object location. Prior to the start of testing, animals received three habituation sessions, in which mice were placed individually into the arena for 5 min. All testing was conducted in plastic, rectangular arenas that measured 38 cm × 40 cm × 40 cm. An overhead camera was used to record animals’ behavior for subsequent analysis. The objects were made of glass, metal, or plastic, and they varied in shape, color, and size, so that they differed markedly between each other. Both the arena and the objects were cleaned between each trial to remove odor cues. The objects used were counterbalanced across phases and, in the test phase, the placement (left or right of arena) of the recent object (temporal order memory test), novel object (novel object recognition test), or displaced object (novel spatial location test) was also counterbalanced among animals. Time spent exploring each object was defined as directing the nose at the object at a distance <1 cm and actively exploring it (i.e., sniffing and/or interacting with the object). Mice were exposed to IA2BC for 16 weeks before testing began; testing occurred beginning at 24 h withdrawal from the last alcohol session and 30 min following guanfacine/vehicle administration. Each session was 5 min in duration. Animals were always placed in the center of the arena at the start of the sessions. The animal was returned to their home cage with its respective cage mate in an adjoining holding area between phases. A few TOM test videos were recorded incorrectly, so data from those mice could not be included. Groups were matched for ethanol intake and body weight, and animals were randomized for dose across tests. Forty-three mice were used for these experiments (same animals for the three tests).

#### Temporal order memory (TOM) test

The TOM test was used to assess recency memory or the ability to retain the memory of an old versus recent object (Barker et al., 2007). This test involved three phases. Sample Phase 1 introduced two identical objects in the arena. Sample Phase 2 introduced two different identical objects. The test phase included two objects, one from each of the sampling phases. There was a 1 h delay between Sample Phases 1 and 2, and a 15 min delay between Sample Phase 2 and Test. If more time is spent exploring the earlier presented object (object from Sample Phase 1), this indicates more intact recency memory. Thirty-eight mice (20 males, 18 females, *n* = 6–7/group) were used in this experiment.

#### Novel object recognition (NOR) test

The NOR test was employed to assess the ability to recognize a novel versus familiar object (Lueptow, 2017). This test involved two phases. In Sample Phase 1, identical objects were presented. There was a 1 h delay between Sample Phase 1 and Test Phase. In the Test Phase, one of the objects was replaced with a novel object. If more time is spent exploring the novel object, this indicates more intact novel object recognition ability. Forty-one mice (21 males, 20 females, *n* = 6–7/group) were used in this experiment.

#### Novel spatial location (NSL) test

The NSL test was employed to assess the ability to recall the old versus new position of an object within the environment (Denninger et al., 2018). This test involved two phases. In Sample Phase 1, two identical objects were presented. There was a 1 h delay between Sample Phase 1 and Test Phase. In the Test Phase, one of the objects was moved in a new position in the arena. If more time is spent exploring the object with the new location, this indicates more intact spatial memory ability. Forty-one mice (23 males, 18 females, *n* = 5–8/group) were used in this experiment.

### Immunohistochemistry (IHC)

#### Brain tissue preparation

A separate cohort of mice was used for immunohistochemistry (IHC); following 8 weeks of IA2BC access, 24 h after the last drinking session, IA2BC and control mice were deeply anesthetized and transcardially perfused with phosphate-buffered saline (PBS) followed by 4% paraformaldehyde (PFA). Brains were removed, postfixed at 4°C for 24 h, and then transferred to 30% sucrose at least until saturation. Brain sections were cut using a cryostat into 30 μm sections and then stored in a cryoprotectant solution at −20°C until processed.

#### Immunohistochemistry protocol

Every sixth section (180 μm apart) for LC (bregma range: −4.96 to −5.68 mm) and NTS (bregma range: −6.24 to −8.24 mm) were processed. Free-floating sections were washed in Tris-buffered saline (TBS) after every incubation. Following initial TBS washes, sodium citrate buffer 10 mM pH 6 antigen retrieval was performed at 80°C for 30 min. Sections were then blocked for 1 h in 3% normal goat serum and 0.3% Triton X-100 in TBS at room temperature (RT) and then transferred into a mouse anti-TH primary antibody (Millipore MAB318, 1:500), and rabbit anti-DeltaFosB primary antibody (Cell Signaling 14695, 1:450) in blocking solution for 24 h at 4°C. Sections were then incubated in secondary antibodies: goat anti-mouse AF594 (Invitrogen A-11032, 1:200) and/or goat anti-rabbit AF488 (Jackson ImmunoResearch 111-545-003, 1:200), in blocking solution for 2 h at RT. Sections were then mounted and coverslipped with mounting medium (VECTASHIELD Antifade Mounting Medium with DAPI, Vector Laboratories).

#### Quantification

The number of TH expressing and DeltaFosB expressing cells was quantified using an Olympus BX-51 microscope equipped with a AxioCam 506m monochrome live video camera (Carl Zeiss), a three-axis MAC6000 XYZ motorized stage (Ludl Electronics), and a personal computer workstation, as previously described (Ferragud et al., 2020, 2021; Minnig et al., 2021). Briefly, either the LC or the NTS were outlined virtually on the digitized image of each section using the Optical Fractionator Workflow module of Stereo Investigator software (MicroBrightField). One hemisphere was randomly chosen for each section; the contours of the areas of interest were drawn using an Olympus UPlanFL *N* × 10 objective with numerical aperture 0.30 and counted using an Olympus UPlanFL *N* × 40 objective with numerical aperture 0.75. For the NTS, positive cells were quantified using the ImageJ Cell Counter plugin. Cell counts were normalized to area and compared across subjects; counts were performed by an experimenter blind to conditions.

### Statistical analysis

Ethanol, water, and sucrose intake were analyzed with repeated-measures three-way ANOVAs with Sex, Dose, and Time as within-subject factors using incremental intake values. Post hoc comparisons were performed using cumulative intake values, which are shown in the figures. Discrimination index in the memory tests was analyzed using a factorial two-way ANOVA with Sex and Dose as between-subjects factors. Data from the IHC studies were analyzed using either two-way ANOVAs (Sex and Group as between-subjects factors) or with Student's *t* tests once sexes were pooled. Post hoc comparisons were performed using either the Dunnett's or the Duncan's test; Student's *t* test was used when only comparing two groups. Mice whose data were >3 SDs from the jackknifed group mean were considered outliers and therefore excluded from further analysis (3 males in the body temperature clonidine/saline experiment, 4 males in the body temperature clonidine/ethanol experiment, 1 male in the loss of righting reflex clonidine experiment, 2 males in the loss of righting reflex guanfacine experiment, 2 males in the guanfacine ethanol drinking experiment, 1 male in the clonidine sucrose drinking experiment, 1 male in the guanfacine sucrose drinking experiment, 1 female in the NTS IHC experiment, 1 female in the NOR test). Significance was set at *p* ≤ 0.05. The software/graphic packages used were StatSoft Statistica 12 and GraphPad Prism 8.

## Results

### Effects of the α2 AR agonists clonidine and guanfacine on body temperature and ethanol-induced loss of righting reflex

**Figure 1.**
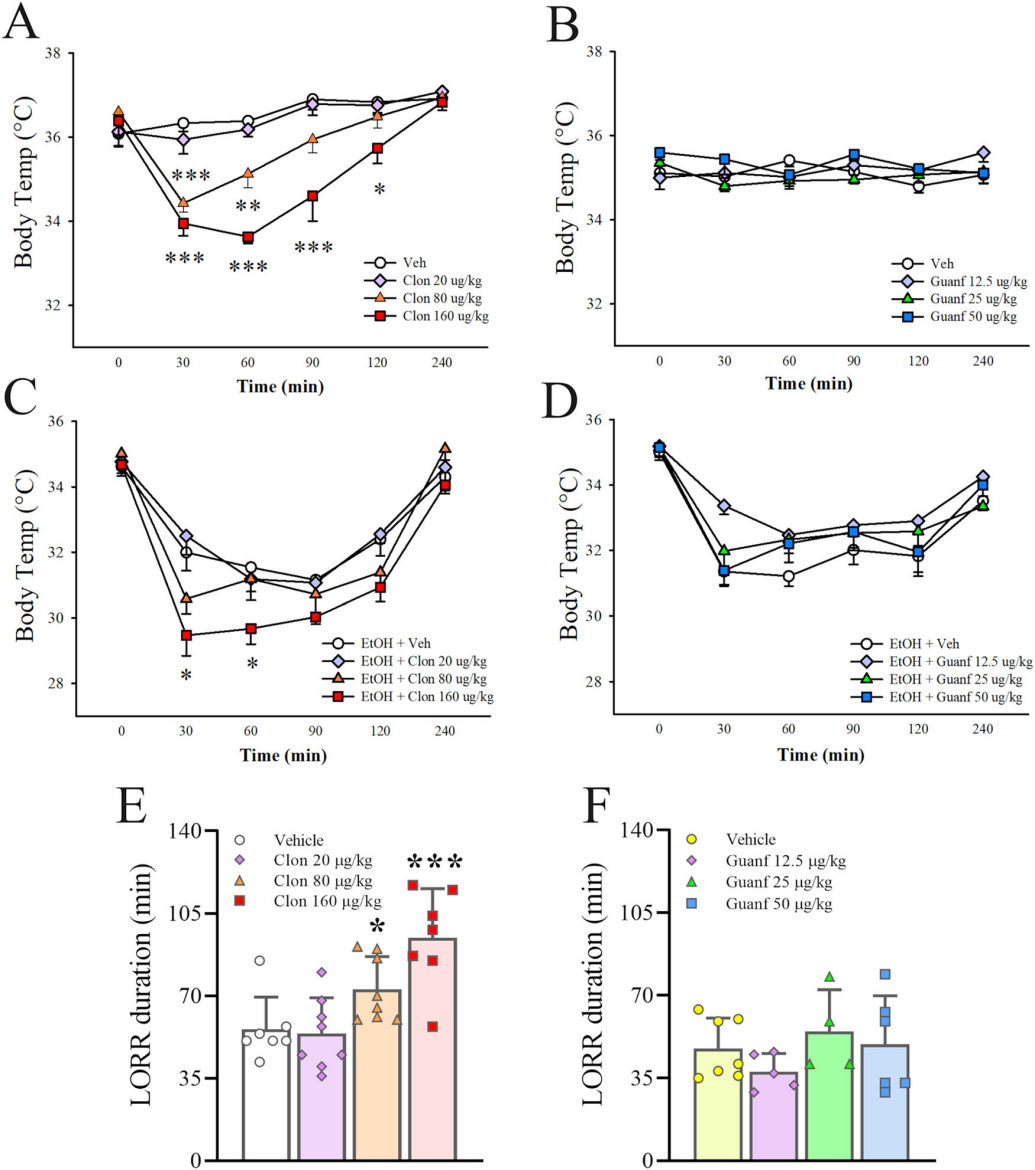
Ethanol-naive male C57Bl/6J mice were treated with either clonidine (Clon, 0–160 µg/kg, i.p.) or guanfacine (Guanf, 0–50 µg/kg, i.p.) and their effect on body temperature were assessed without ethanol (after saline only; ***A***, ***B***) or with ethanol (3.6 g/kg, i.p.; ***C***, ***D***). Clonidine and guanfacine effects were also assessed on ethanol-induced (3.6 g/kg, i.p.) loss of righting reflex (LORR; ***E***, ***F***). While clonidine reduced body temperature on its own as well as in combination with ethanol and it increased the duration of LORR, guanfacine did not affect these measures. Please note the different *y*-axis scale between panels ***A***, ***B*** (no ethanol) and panels ***C***, ***D*** (with ethanol). Data represent mean ± SEM (*n* = 6–8/group body temp; *n* = 4–8/group LORR). *p ≤ 0.05, **p ≤ 0.01, ***p ≤ 0.001 versus Vehicle (Duncan's test).

The α2 AR agonists clonidine and guanfacine were tested for their ability to affect body temperature in ethanol-naive C57Bl/6J mice. Each agonist was tested by itself first, and in combination with ethanol then (20% v/v in saline, i.p.). Drugs were administered 30 min prior to ethanol (or vehicle), and body temperature was measured rectally at different time points. A three-way ANOVA revealed that clonidine (0, 20, 80, 160 µg/kg, i.p.), as shown in Figure 1*A*,*C*, dose-dependently reduced body temperature differentially across time, both when administered alone and when administered before ethanol (Dose: *F*_(3,46)_ = 16.0, *p* ≤ 0.00001; Dose × Time: *F*_(12,184)_ = 3.9, *p* = 0.00002; Dose × EtOH: *F*_(3,46)_ = 0.2, *p* = 0.88, not significant (n.s.); Dose × EtOH × Time: *F*_(12,184)_ = 1.1, *p* = 0.34, n.s.). Since technically data could not be analyzed using a three-way ANOVA since the Ethanol and No Ethanol conditions were run on two different days, two-way ANOVAs were performed and they confirmed that clonidine affected body temperature dose-dependently in the absence of ethanol (Dose: *F*_(3,22)_ = 22.8, *p* ≤ 0.00001; Dose × Time: *F*_(12,88)_ = 4.1, *p* = 0.00004). Indeed, clonidine administration significantly reduced body temperature at all time points tested, according to one-way ANOVAs, except at 240 min, when the temperature went back to basal levels. Clonidine was most effective at the 30 and 60 min time points.

In presence of ethanol, clonidine further reduced body temperature (Dose: *F*_(3,24) _= 4.80, *p* = 0.0093; Dose × Time: *F*_(12,96) _= 1.92, *p* = 0.041), showing that clonidine potentiated ethanol-induced hypothermia. The effect was significant at the 30 and 60 min time points. No deaths were observed. Even though the “no ethanol” and “ethanol” tests were run at different times (Fig. 1*A*,*C*) and, therefore, no direct statistical comparisons can be made between the two, the basal temperature levels before any drug administration did not differ between the two groups (group means 36.1 and 36.4°C), while the ethanol group showed lower temperatures under vehicle conditions compared with the “no ethanol” group (group mean difference ethanol/vehicle vs no ethanol/vehicle group, 0 min: −2.4°C, 30 min: −2.8°C, 60 min: −2.3°C, 90 min: −1.1°C, 120 min: −0.1°C, 240 min: 0.5°C).

The three-way ANOVA revealed that guanfacine (0, 12.5, 25, 50 µg/kg), on the other hand, did not affect body temperature, either per se or in combination with ethanol (Dose: *F*_(3,46)_ = 2.3, *p* = 0.89, n.s.; Dose × Time: *F*_(12,184)_ = 1.1, *p* = 0.39, n.s.; Dose × EtOH: *F*_(3,46)_ = 1.6, *p* = 0.21, n.s.; Dose × EtOH × Time: *F*_(12,184)_ = 2.2, *p* = 0.012), as shown in Figure 1*B*,*D*. Since technically data could not be analyzed using a three-way ANOVA since the Ethanol and No Ethanol conditions were run on two different days, two-way ANOVAs were performed and confirmed that guanfacine did not affect body temperature either in the absence of ethanol (Dose: *F*_(3,23)_ = 1.4, *p* = 0.26, n.s.; Dose × Time: *F*_(12,92)_ = 1.4, *p* = 0.19, n.s.) or in the presence of ethanol (Dose: *F*_(3,23)_ = 2.1, *p* = 0.13, n.s.; Dose × Time: *F*_(12,92)_ = 1.8, *p* = 0.063, n.s.).

In the animals where either clonidine or guanfacine were tested in the presence of ethanol, latency to and duration of the ethanol-induced loss of righting reflex (LORR) was measured at the same time. Data analysis showed that clonidine dose-dependently potentiated LORR duration, as shown in Figure 1*E* (*F*_(3,26)_ = 10.11, *p* = 0.0001); the middle and highest dose of clonidine (80 and 160 µg/kg) both significantly increased LORR duration, by 30.5 and 69.5%, respectively. Guanfacine, on the other hand, had no effect on LORR duration, as shown in Figure 1*F* (*F*_(3,18)_ = 0.98, *p* = 0.42, n.s.). LORR latency was unaffected by either clonidine (*F*_(3,26)_ = 0.15, *p* = 0.93, n.s.) or guanfacine (*F*_(3,18)_ = 0.08, *p* = 0.97, n.s.; data not shown).

### Effects of the α2 AR agonist clonidine on ethanol intake

**Figure 2.**
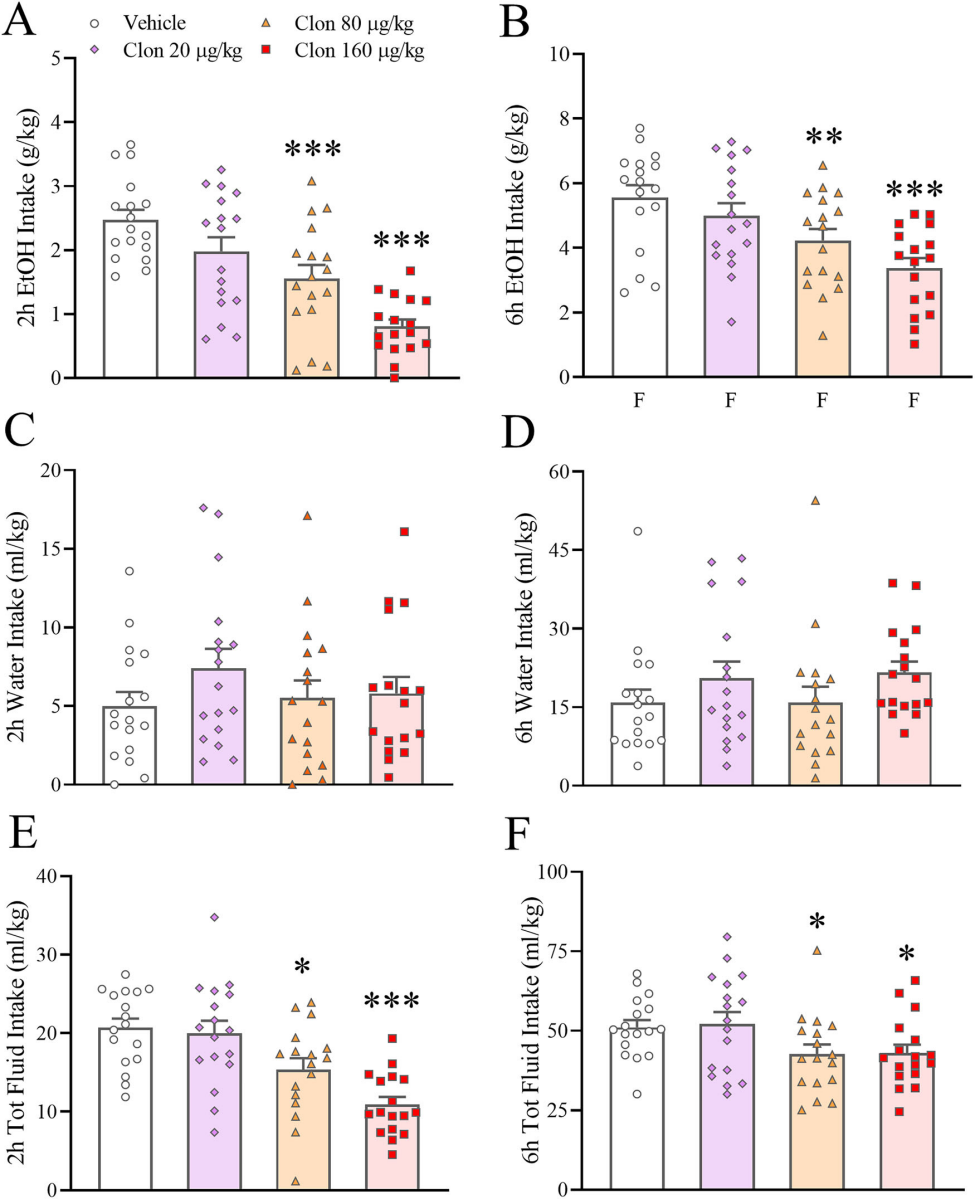
Male and female C57Bl/6J mice were exposed to intermittent access to alcohol (IA2BC, Monday, Wednesday, Friday, 24 h/d, 20% v/v ethanol vs water) for at least 12 weeks before testing began. Administration of Clonidine (Clon, 0–160 µg/kg, i.p.) dose-dependently reduced 2 and 6 h ethanol intake (***A***, ***B***). While clonidine did not affect water intake (***C***, ***D***), it reduced 2 and 6 h total fluid intake (***E***, ***F***). Data represent mean ± SEM (in each sex, *n* = 8–9/group). *p ≤ 0.05, **p ≤ 0.01, ***p ≤ 0.001 versus Vehicle (Dunnett’s test). Extended Data Figure 2-1 shows the data relative to the effect of Clonidine on ethanol intake disaggregated by sex.

We found a statistically significant and dose-dependent effect of clonidine (0–160 µg/kg, i.p.) on 2 and 6 h ethanol intake in C57Bl/6J mice subject to an IA2BC paradigm, which was independent from sex (Dose: *F*_(3,45)_ = 11.87, *p* ≤ 0.00001; Dose × Sex: *F*_(3,45)_ = 2.11, *p* = 0.11, n.s.; Dose × Time: *F*_(3,45)_ = 2.25, *p* = 0.09, n.s.; Dose × Time × Sex: *F*_(3,45)_ = 1.00, *p* = 0.40, n.s.). Post hoc analysis showed that the 80 µg/kg and the 160 µg/kg doses both significantly reduced 2 and 6 h ethanol intake; the 80 µg/kg dose caused reductions of 37.1 and 23.9%, and the 160 µg/kg dose reductions of 67.4 and 39.4%, at the 2 and 6 h time point, respectively (Fig. 2*A*,*B*). Water intake was not significantly affected by clonidine (Fig. 2*C*,*D*; Dose: *F*_(3,45)_ = 1.44, *p* = 0.25, n.s.; Dose × Sex: *F*_(3,45)_ = 0.61, *p* = 0.61, n.s.; Dose × Time: *F*_(3,45)_ = 1.16, *p* = 0.33, n.s.; Dose × Time × Sex: *F*_(3,45)_ = 0.83, *p* = 0.48, n.s.). Total fluid intake was significantly affected by clonidine, as shown in Figure 2*E*,*F* (Dose: *F*_(3,45)_ = 4.61, *p* = 0.007; Dose × Sex: *F*_(3,45)_ = 1.79, *p* = 0.16, n.s.; Dose × Time: *F*_(3,45)_ = 2.83, *p* = 0.049; Dose × Time × Sex: *F*_(3,45)_ = 0.34, *p* = 0.80, n.s.); the 80 µg/kg and the 160 µg/kg doses significantly reduced total fluid intake mainly at 2 h (25.7 and 47.2% reduction, respectively) but also at 6 h. Extended Data Figure 2-1 shows the intake data disaggregated by sex. In this experiment, the mean ± SEM 24 h cumulative ethanol intake in male and females under vehicle conditions was 9.56 ± 1.09 and 12.44 ± 1.05 g/kg, respectively. The effects of clonidine on ethanol intake and preference did not persist until the 24 h time point (data not shown).

10.1523/ENEURO.0368-25.2026.f2-1 Figure 2-1 Male and female C57Bl/6J mice exposed to intermittent access to alcohol (IA2BC) were administered Clonidine (Clon, 0-160 µg/kg, i.p.). Data are here reported disaggregated by sex: **(A, C, E)** males, **(B, D, F)** females. (**A**, **B**) 2 h and 6 h ethanol intake, (**C**, **D**) 2 h and 6 h water intake, (**E**, **F**) 2 h and 6 h total fluid intake. Data represent Mean ± SEM (in each sex, *n* = 8-9/group). Download Figure 2-1, TIF file.

### Effects of the α2 AR agonist guanfacine on ethanol intake

**Figure 3.**
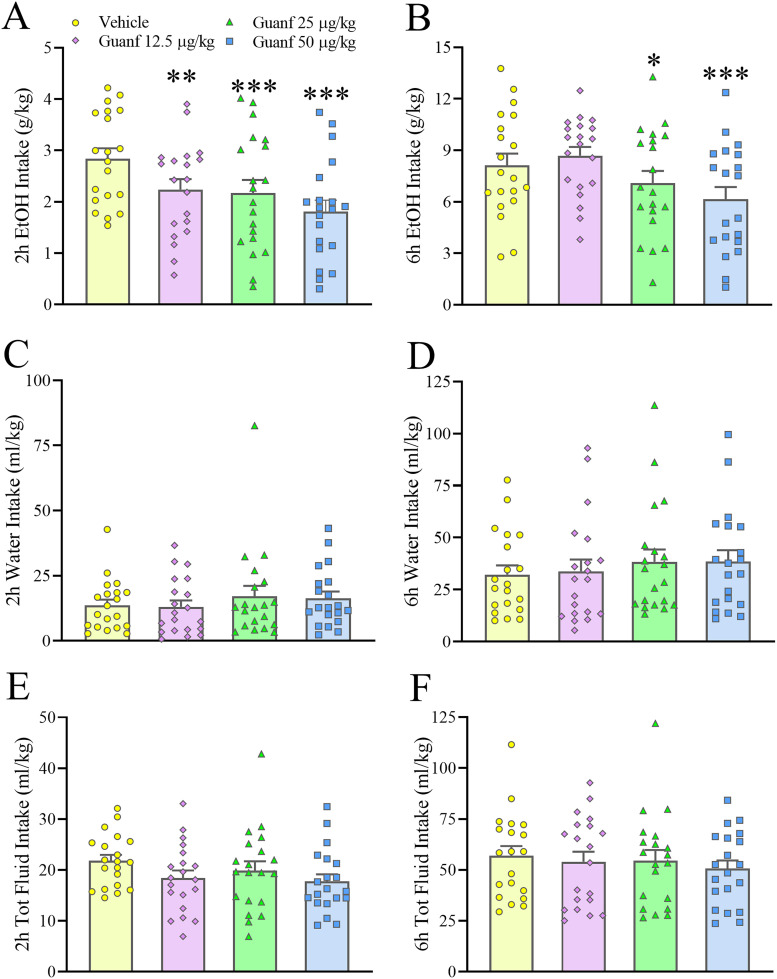
Male and female C57Bl/6J mice were exposed to intermittent access to alcohol (IA2BC) for at least 12 weeks before testing began. Administration of guanfacine (Guan, 0–50 µg/kg, i.p.) dose-dependently reduced 2 and 6 h ethanol intake (***A***, ***B***). Guanfacine did not affect water intake (***C***, ***D***) or total fluid intake (***E***, ***F***). Data represent mean ± SEM (in each sex, *n* = 9–11/group). *p ≤ 0.05, **p ≤ 0.01, ***p ≤ 0.001 versus Vehicle (Dunnett’s test). Extended Data Figure 3-1 shows the data relative to the effect of guanfacine on ethanol intake disaggregated by sex.

Guanfacine (0–50 µg/kg, i.p.) significantly and dose-dependently affected IA2BC ethanol intake, independently from sex (Dose: *F*_(3,54)_ = 7.75, *p* = 0.0002; Dose × Sex: *F*_(3,54)_ = 0.74, *p* = 0.53, n.s.; Dose × Time: *F*_(3,54)_ = 0.81, *p* = 0.49, n.s.; Dose × Time × Sex: *F*_(3,54)_ = 1.11, *p* = 0.35, n.s.). Post hoc analysis showed that all three doses of guanfacine (12.5, 25, and 50 µg/kg) significantly reduced ethanol intake at the 2 h time point, and the middle and high dose at the 6 h time point; the highest dose, 50 µg/kg, caused reductions of 35.9 and 25.9% at the 2 and 6 h time point, respectively (Fig. 3*A*,*B*). Water intake was not significantly affected by guanfacine treatment (Fig. 3*C*,*D*; Dose: *F*_(3,54)_ = 1.02, *p* = 0.39, n.s.; Dose × Sex: *F*_(3,54)_ = 0.25, *p* = 0.86, n.s.; Dose × Time: *F*_(3,54)_ = 0.04, *p* = 0.99, n.s.; Dose × Time × Sex: *F*_(3,54)_ = 1.02, *p* = 0.39, n.s.). Total fluid intake was also unaffected, as shown in Figure 3*E*,*F* (Dose: *F*_(3,54)_ = 1.07, *p* = 0.37, n.s.; Dose × Sex: *F*_(3,54)_ = 0.64, *p* = 0.59, n.s.; Dose × Time: *F*_(3,54)_ = 0.38, *p* = 0.77, n.s.; Dose × Time × Sex: *F*_(3,54)_ = 0.19, *p* = 0.90, n.s.). Extended Data Figure 3-1 shows the intake data disaggregated by sex. In this experiment, the mean ± SEM 24 h cumulative ethanol intake in male and females under vehicle conditions was 8.09 ± 0.66 and 26.45 ± 2.35 g/kg, respectively. The effects of guanfacine on ethanol intake and preference did not persist until the 24 h time point (data not shown).

10.1523/ENEURO.0368-25.2026.f3-1 Figure 3-1 Male and female C57Bl/6J mice exposed to intermittent access to alcohol (IA2BC) were administered Guanfacine (Guan, 0-50 µg/kg, i.p.). Data are here reported disaggregated by sex: **(A, C, E)** males, **(B, D, F)** females. (**A**, **B**) 2 h and 6 h ethanol intake, (**C**, **D**) 2 h and 6 h water intake, (**E**, **F**) 2 h and 6 h total fluid intake. Data represent Mean ± SEM (in each sex, *n* = 8-9/group). Download Figure 3-1, TIF file.

### Effects of the α2 AR agonists clonidine and guanfacine on sucrose intake

**Figure 4.**
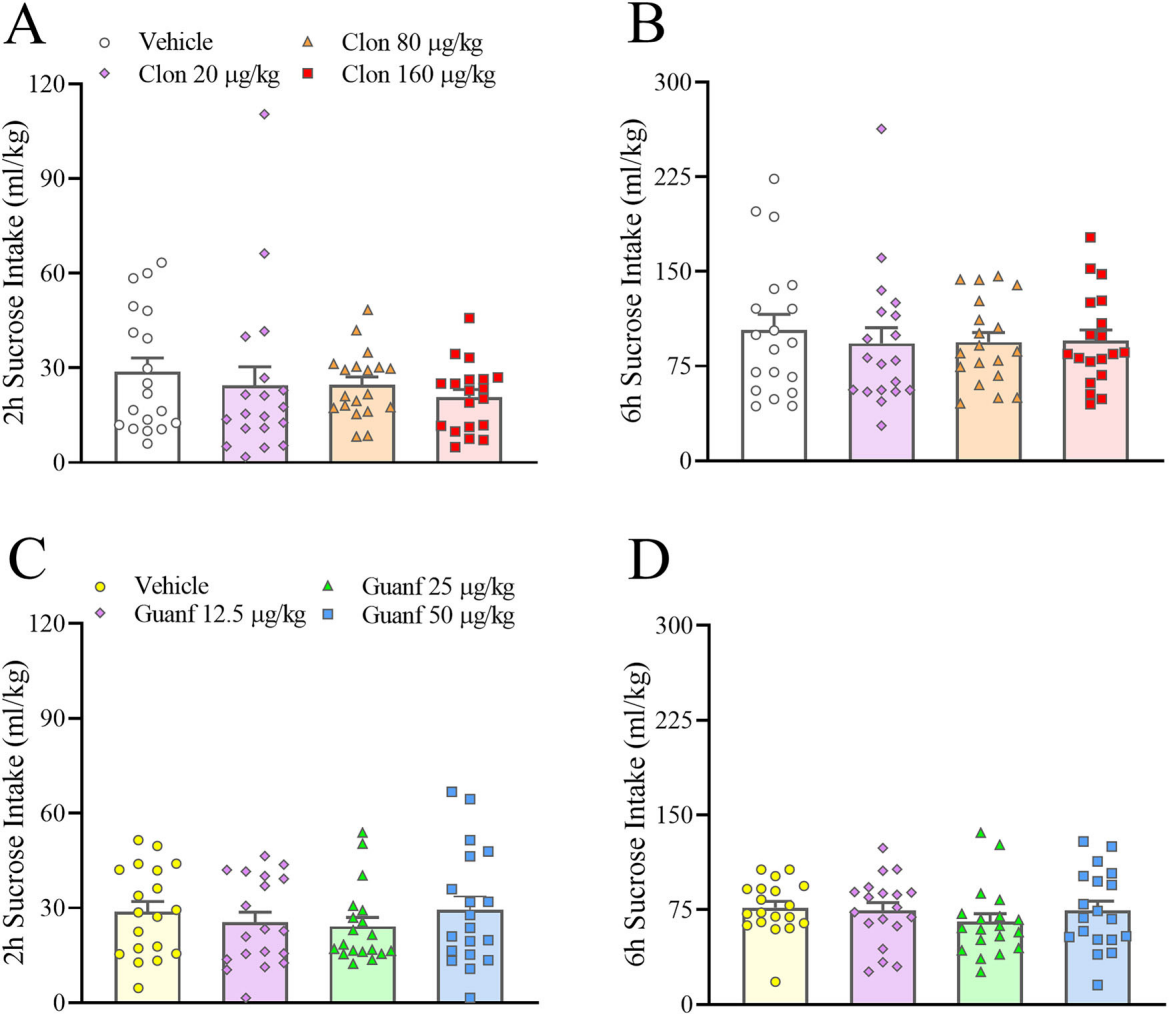
Male and female C57Bl/6J mice were exposed to intermittent access to sucrose (Monday, Wednesday, Friday, 24 h/d, 1.15% w/v sucrose vs water) for at least 7 weeks before drug testing began. Neither clonidine (Clon, 0–160 µg/kg, i.p.; ***A***, ***B***) nor guanfacine (Guan, 0–50 µg/kg, i.p.; ***C***, ***D***) had an effect on 2 and 6 h sucrose intake. Data represent mean ± SEM (in each sex, *n* = 9–10/group Clon, *n* = 8–11/group Guanf).

Clonidine (0–160 µg/kg, i.p.) did not significantly affect sucrose intake in an IA2BC paradigm at the 2 and 6 h time points, as shown in Figure 4*A*,*B* (Dose: *F*_(3,51)_ = 0.55, *p* = 0.65, n.s.; Dose × Sex: *F*_(3,51)_ = 1.06, *p* = 0.37, n.s.; Dose × Time: *F*_(3,51)_ = 0.79, *p* = 0.51, n.s.; Dose × Time × Sex: *F*_(3,51)_ = 0.99, *p* = 0.40, n.s.). Guanfacine (0–50 µg/kg, i.p.) also did not affect sucrose intake, as shown in Figure 4*C*,*D* (Dose: *F*_(3,51)_ = 1.10, *p* = 0.36, n.s.; Dose × Sex: *F*_(3,51)_ = 0.25, *p* = 0.86, n.s.; Dose × Time: *F*_(3,51)_ = 0.97, *p* = 0.42, n.s.; Dose × Time × Sex: *F*_(3,51)_ = 0.33, *p* = 0.80, n.s.). Twenty-four hour sucrose intake and concurrent water intake were also not affected by either agonist (data not shown).

### Effects of the α2 AR agonist guanfacine on ethanol-induced cognitive deficits

Mice were exposed to IA2BC for 16 weeks before being tested for the ability of guanfacine (0–150 µg/kg, i.p.) to affect cognitive performance, and testing occurred at 24 h withdrawal from the last alcohol session, 30 min following drug administration. Mean ± SEM of the 24 h cumulative ethanol intake during the last three sessions was 11.5 ± 1.1 g/kg (males) and 17.6 ± 0.8 g/kg (females). Three tests of object recognition memory were used, each requiring judgments about either recency (temporal order memory test), object identity (novel object recognition test), or object location (novel spatial location test). The discrimination index was calculated as the ratio between the time spent exploring the recent/new/novel location object and the total exploration time in the Test phase.

#### Temporal order memory (TOM) test

**Figure 5.**
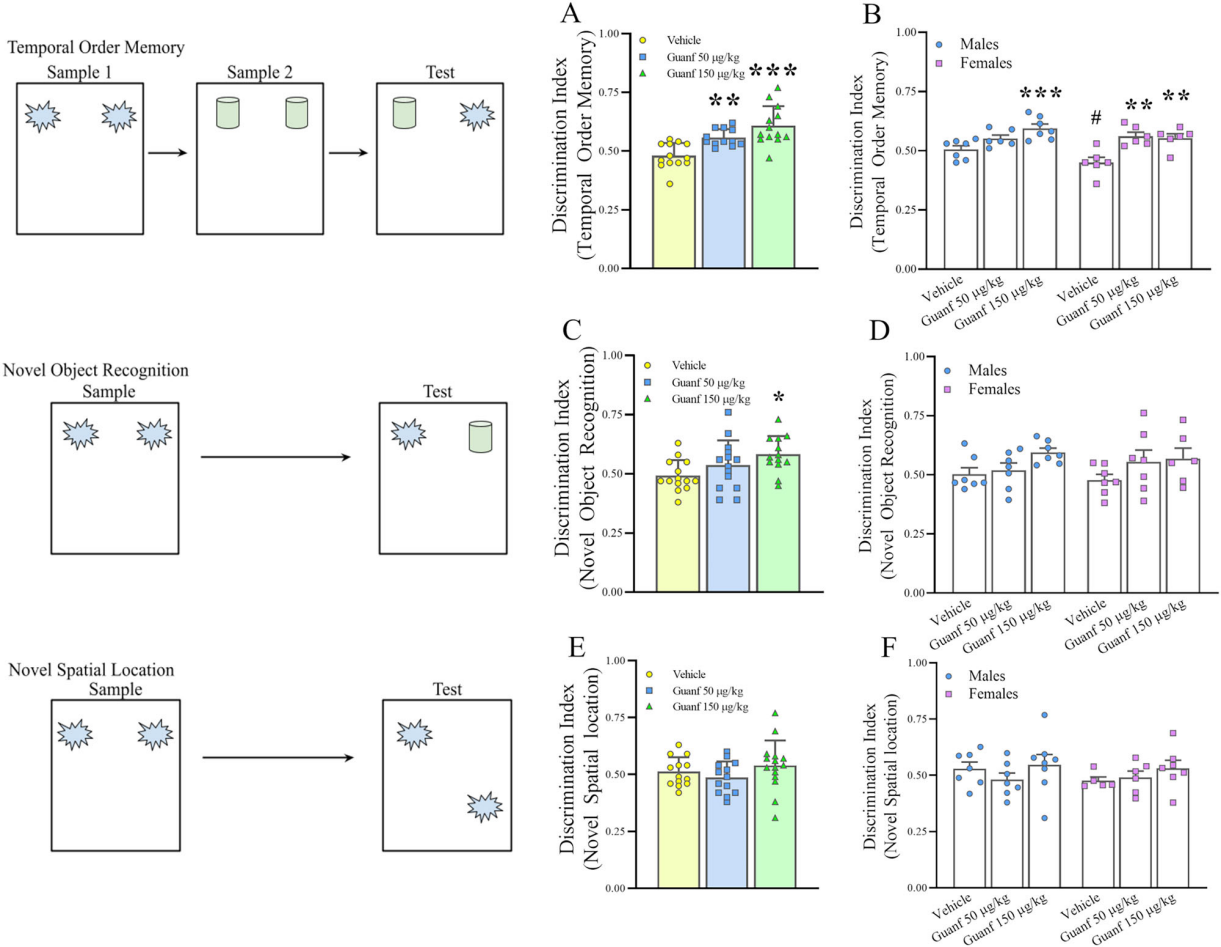
Male and female C57Bl/6J mice were exposed to intermittent access to alcohol (IA2BC) for 16 weeks before testing began. Administration of Guanfacine (0–150 µg/kg, i.p.) dose-dependently improved performance in (***A***) a temporal order memory (TOM) test and (***C***) in a novel object recognition (NOR) test, while it had no effect on performance in (***E***) a novel spatial location (NSL) test. ***B***, ***D***, ***F***, Data disaggregated by sex. Schemes on the left depict the design of each test. Data represent mean ± SEM (in each sex, *n* = 5–8/group). *p ≤ 0.05, **p ≤ 0.01, ***p ≤ 0.001 versus Vehicle, ^#^*p* ≤ 0.05 versus Males Vehicle (Newman–Keuls test).

As shown in Figure 5*A*, guanfacine treatment significantly affected the discrimination index in the TOM test, differentially between sexes (Dose: *F*_(2,32)_ = 18.74, *p* ≤ 0.00001; Dose × Sex: *F*_(2,32)_ = 3.48, *p* = 0.04), suggesting that the drug improved the ability of mice to recognize the recency of the objects. As shown in Figure 5*B*, when data from males and females were analyzed segregated, guanfacine increased the index more potently in female mice, since both doses significantly differed from vehicle, while in males only the highest dose was statistically different from vehicle; the middle dose increased the discrimination index by 24.6% in female mice, but only by 8.6% in males, while the highest doses had comparable effects in the two sexes (23.1, 28.2%). In addition, under vehicle conditions, female mice showed a lower discrimination index than males (*p* = 0.05), suggesting a larger impairment of females in this test. When data from males and females were pooled together to be able to compare with the other cognitive tests, post hoc analyses showed that both the middle (50 µg/kg) and the highest (150 µg/kg) dose significantly increased the ability of mice to recognize the recent object, compared with vehicle (Fig. 5*A*).

#### Novel object recognition (NOR) test

As shown in Figure 5*C*, although not very efficiently, guanfacine treatment significantly affected the discrimination index in the NOR test, suggesting that the drug slightly improved the ability of mice to recognize the identity of the objects; the effect was independent from sex (Dose: *F*_(2,41)_ = 4.30, *p* = 0.02; Dose × Sex: *F*_(2,41)_ = 0.67, *p* = 0.52, n.s.). Post hoc analyses showed that only the highest dose (150 µg/kg) significantly increased the ability of mice to recognize the novel object (18.8% increase in discrimination index; Fig. 5*C*). When the data were segregated between sexes, no significant differences were found following post hoc analysis (Fig. 5*D*).

#### Novel spatial location (NSL) test

As shown in Figure 5*E*, guanfacine treatment did not significantly affect the discrimination index in the NSL test (Dose: *F*_(2,35)_ = 1.36, *p* = 0.27, n.s.; Dose × Sex: *F*_(2,35)_ = 0.43, *p* = 0.65, n.s.), showing that the drug did not change the ability of mice to recognize the location of the objects.

### Chronic intermittent ethanol drinking increases the number of activated TH expressing cells in the LC and NTS

**Figure 6.**
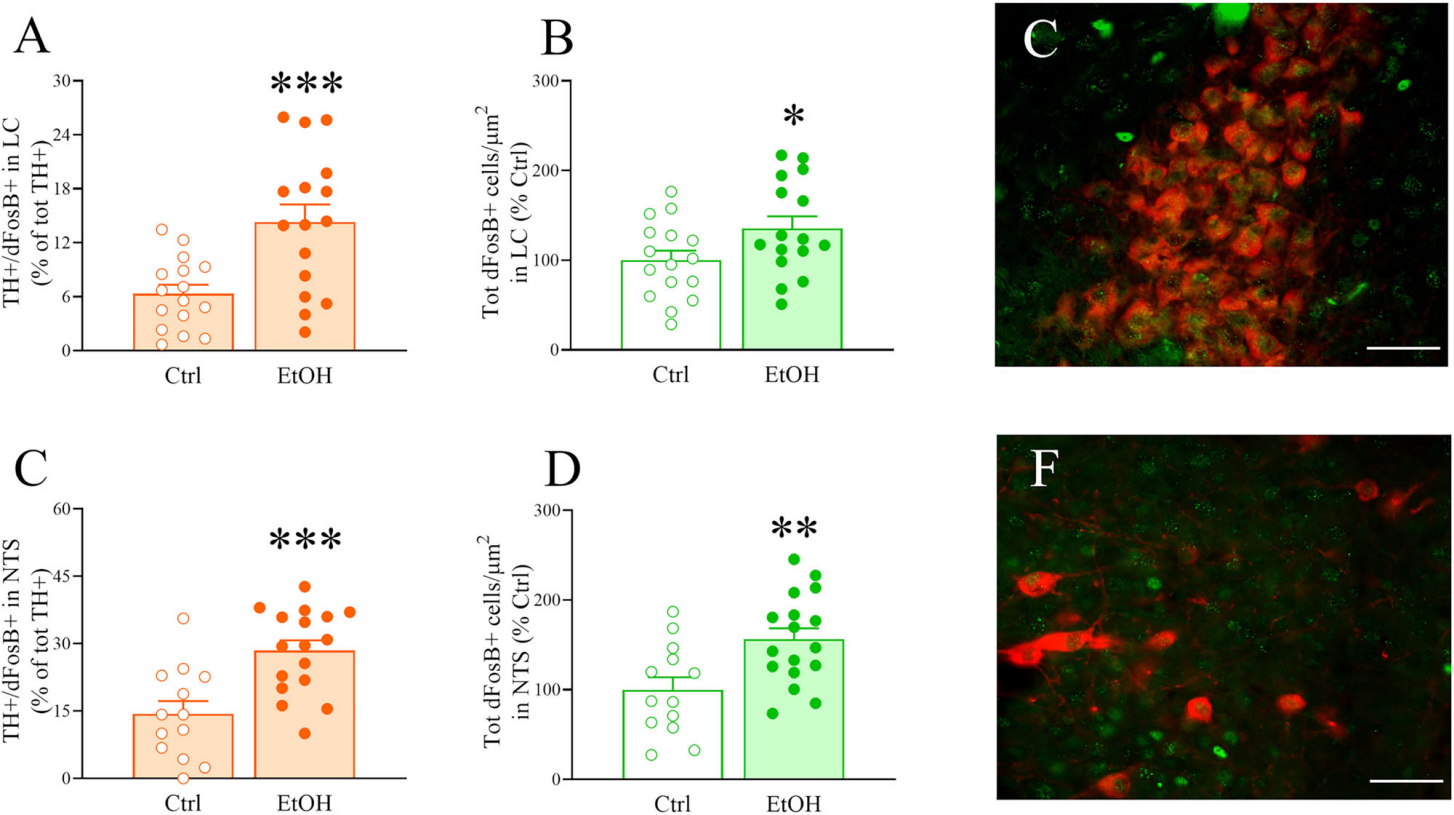
Male and female C57Bl/6J mice were exposed to intermittent access to alcohol (IA2BC) for 8 weeks and were then killed for immunofluorescence 24 h after the end of the last drinking session. ***A***, The number of cells immunoreactive for both TH and dFosB is elevated in the LC of male and female mice exposed to intermittent access to alcohol (IA2BC, “EtOH”), compared with control, water only mice (“Ctrl”). ***B***, Total dFosB expressing cells is also increased in EtOH mice in LC, compared with Ctrl. Data are reported with the two sexes pooled. ***C***, Representative TH and dFosB staining in LC (20× magnification; scale bar, 100 µm; red, TH; green, dFosB). ***D***, The number of cells immunoreactive for both TH and dFosB is elevated in the NTS of male and female EtOH C57Bl/6J mice, compared with Ctrl. ***E***, Total dFosB expressing cells is also increased in the NTS of EtOH mice, compared with Ctrl. Data are reported with the two sexes pooled. ***F***, Representative TH and dFosB staining in NTS (20× magnification; scale bar, 100 µm; red, TH; green, dFosB). Data represent mean ± SEM (*n* = 4–9/group). *p ≤ 0.05, **p ≤ 0.01, ***p ≤ 0.001 versus Ctrl (Student's *t* test).

After 8 weeks of IA2BC access, IA2BC and control mice were killed 24 h after the last drinking session. Brain slices containing the NEergic nuclei LC and NTS were subject to fluorescence immunohistochemistry (IHC) for tyrosine hydroxylase (TH), a sensitive marker of catecholaminergic neurons, and DeltaFosB (dFosB in figure labels), a neuronal marker of long-lasting changes in neuronal activation; fluorescent staining was quantified by cell counting. Mean ± SEM ethanol intake during the last sessions was as follows: 14.8 ± 1.3 g/kg (males); 19.8 ± 1.6 g/kg (females; data not shown). As shown in Figure 6*A*, mice with a history of chronic, intermittent alcohol drinking displayed a higher number of cells coexpressing TH and DeltaFosB in the LC, independently from sex (Ethanol: *F*_(1,28)_ = 12.52, *p* = 0.0014; Ethanol × Sex: *F*_(1,28)_ = 0.42, *p* = 0.52, n.s.); when sexes were pooled together, IA2BC overall caused a 125.8% increase in LC cells expressing both TH and DeltaFosB (Student's *t* test: *t*_(30)_ = 3.66, *p* = 0.001). As shown in Figure 6*B*, when looking at the total number of cells expressing DeltaFosB in the LC, the two-way ANOVA showed a strong trend (n.s.) for an effect of alcohol, which was again independent from sex (Ethanol: *F*_(1,28)_ = 3.87, *p* = 0.059, n.s.; Ethanol × Sex: *F*_(1,28)_ = 0.69, *p* = 0.41, n.s.); when sexes were pooled together, IA2BC overall caused a 35.6% increase in LC cells expressing DeltaFosB (Student's *t* test: *t*_(30)_ = 2.09, *p* ≤ 0.05). IA2BC, on the other hand, did not affect the total number of cells expressing TH in the LC (Ethanol: *F*_(1,28)_ = 0.13, *p* = 0.72, n.s.; Ethanol × Sex: *F*_(1,28)_ = 1.28, *p* = 0.27, n.s., data not shown). Figure 6*C* shows representative TH (red) and DeltaFosB (green) staining in LC.

Similarly, as shown in Figure 6*D*, in the NTS mice with a history of chronic, intermittent alcohol drinking displayed a higher number of cells that coexpress TH and DeltaFosB, independently from sex (Ethanol: *F*_(1,26)_ = 13.79, *p* ≤ 0.001; Ethanol × Sex: *F*_(1,26)_ = 0.03, *p* = 0.86, n.s.); IA2BC overall caused a 97.8% increase in NTS cells expressing both TH and DeltaFosB (Student's *t* test: *t*_(28)_ = 3.91, *p* = 0.0005). As shown in Figure 6*E*, when looking at the total number of cells expressing DeltaFosB in the NTS, alcohol also had a significant effect, which was again independent from sex (Ethanol: *F*_(1,26)_ = 8.20, *p* = 0.008; Ethanol × Sex: *F*_(1,26)_ = 0.27, *p* = 0.61, n.s.); IA2BC overall caused a 56.4% increase in NTS cells expressing DeltaFosB (Student's *t* test: *t*_(28)_ = 2.05, *p* = 0.0049). IA2BC did not affect the total number of cells expressing TH in the NTS (Ethanol: *F*_(1,26)_ = 2.44, *p* = 0.13, n.s.; Ethanol × Sex: *F*_(1,26)_ = 0.66, *p* = 0.42, n.s., data not shown). Figure 6*F* shows representative TH (red) and DeltaFosB (green) staining in NTS.

## Discussion

The main findings of this series of experiments were as follows: (1) The α2 adrenergic receptor (AR) agonist clonidine reduces body temperature per se and potentiates ethanol-induced hypothermia as well as sedative effects; (2) the more selective α2 adrenergic agonist guanfacine does not affect body temperature nor does it potentiate ethanol-induced hypothermia or sedation; (3) both clonidine and guanfacine reduce heavy alcohol drinking but guanfacine does so at lower doses; (4) neither clonidine nor guanfacine reduce sucrose drinking; (5) guanfacine improves cognitive performance in a temporal order test and, less efficiently, in a novel object recognition test, in ethanol drinking mice; and (6) chronic intermittent ethanol drinking increases the number of persistently activated TH positive neurons in the noradrenergic nuclei LC and NTS.

The imidazoline derivative α2 AR agonist clonidine is an old antihypertensive drug today used for several other indications, including the treatment of physical signs of withdrawal from opiates (Gold et al., 1978; Washton and Resnick, 1980; Gossop, 1988; Opitz, 1990). Clonidine can also effectively attenuate various symptoms of alcohol withdrawal in humans and in animal models (Bjorkqvist, 1975; Parale and Kulkarni, 1986; Cushman, 1987) and so do other α2 AR agonists (e.g., lofexidine, dexmedetomidine, guanfacine; Shearman et al., 1980; Riihioja et al., 1997; Gowing et al., 2016). Unfortunately, clonidine has important side effects, including sedation, hypotension, and hypothermia (Laverty, 1970; Delbarre and Schmitt, 1971; Tsoucaris-Kupfer and Schmitt, 1972; Drew et al., 1979; McLennan, 1981; Kumar et al., 2014). Guanfacine has a 20 year safety record in humans (Sorkin and Heel, 1986) and is approved for the treatment of ADHD and tics in children (Arnsten et al., 1988; Chappell et al., 1995; Hunt et al., 1995; Horrigan and Barnhill, 1996; Scahill et al., 2001, 2006, 2015; Boon-yasidhi et al., 2005; Connor et al., 2014). Both clonidine and guanfacine are currently contraindicated with alcohol use, for concerns that it may potentiate its sedative and hypothermic effects, but studies that directly test these potential additive effects with ethanol are very scarce. Our data indeed show that, in C57Bl/6J mice, clonidine dose-dependently reduces body temperature; importantly, clonidine not only induced hypothermia per se but also worsened ethanol-induced hypothermia. In addition, we found that clonidine potentiated the sedative effect of ethanol, measured as an increase in the duration of the loss of righting reflex, at the same doses which we will later observe reduce heavy ethanol drinking (80 and 160 µg/kg). Interestingly, here we observed that, unlike clonidine, guanfacine did not induce, nor did it potentiate, hypothermia or the sedative effects of alcohol at doses that reduce heavy ethanol intake (12.5, 25, and 50 µg/kg). Our current results, therefore, appear to suggest that guanfacine, but not clonidine, could be used safely with alcohol in these regards. In line with our results showing the lack of hypothermic and sedative effects of low doses of guanfacine, previous studies have shown that, in rodents, doses of guanfacine higher than 1,000 µg/kg (1 mg/kg) are required to observe any hypothermic or sedative effects (Van der Laan et al., 1985; Danysz et al., 1987; Minor et al., 1989; Fredriksson et al., 2015). The preclinical data also are in agreement with data obtained in nonhuman primates as well as with clinical observations that low doses of guanfacine, currently used for ADHD, are not sedating and weakly or not at all lower blood pressure (Sorkin and Heel, 1986; Arnsten et al., 1988; Fischetti et al., 1994). The adverse effects induced by clonidine (i.e., sedation, hypothermia, and hypotension) are thought to be due to its lack of binding selectivity (Buccafusco et al., 1984; Kumar et al., 2014), such as actions on thalamic α2B AR (Buzsaki et al., 1991), presynaptic α2A and α2C autoreceptors in the LC and in axon terminals throughout the brain (Berridge and Foote, 1991, 1996), and imidazoline I1 receptor (Coupry et al., 1989; Uhlen and Wikberg, 1991; Uhlen et al., 1994). Guanfacine, on the other hand, is highly selective for α2A, displaying 15–60× higher affinity for the α2A over the α2B and α2C receptor subtypes (Uhlen and Wikberg, 1991; Uhlen et al., 1994), and has a strong affinity toward postsynaptic α2A receptors, having 10× less impact on presynaptic α2A AR, compared with clonidine (Engberg and Eriksson, 1991). Therefore, the lack of sedation and hypothermia following guanfacine administration is likely to be due its weaker actions at α2B and α2C ARs, I1 receptors, and presynaptic α2A (Engberg and Eriksson, 1991; Scahill et al., 2001).

While the effectiveness of α2 AR agonists in relieving opiate and alcohol physical signs of withdrawal has been extensively reported, their effect on drug/alcohol consumption and urge to drink is less established. Here, we used a mouse model of heavy drinking, the intermittent access two bottle choice paradigm (Wise, 1973), which yields high levels of ethanol consumption and long-lasting behavioral and molecular adaptations (Carnicella et al., 2008, 2014; Simms et al., 2008; Hwa et al., 2011; Zhou et al., 2017; Newman et al., 2018; Bloodgood et al., 2021; Quadir et al., 2021a,b; Lepeak et al., 2023). We observed that mice treated with clonidine displayed a reduction of alcohol intake at the two highest doses tested, as well as a reduction of total fluid intake. We also observed that the more selective α2 AR agonist guanfacine reduced ethanol intake at all doses tested, without affecting total fluid intake. The current observation that clonidine and guanfacine, the latter at lower doses that do not induce sedation or hypothermia, reduce voluntary ethanol consumption in a mouse model of heavy drinking indicates that the activation of α2 AR is able to blunt excessive ethanol intake. The effect on ethanol was selective, as sucrose solution intake or concurrent water intake were not affected, suggesting that neither drug produces malaise-like effects that nonspecifically cause a reduction in intake of all palatable solutions. These results are significant and in line with some older studies in alcohol-preferring rats which showed that α2 AR agonists can reduce alcohol intake. Opitz showed that clonidine (75, 150 µg/kg, s.c. or p.o.), guanfacine, and tiamenidine (1,500 µg/kg, p.o.) diminish alcohol consumption in Finnish alcohol-preferring rats (Opitz, 1990); furthermore, clonidine (40, 80 μg/kg, i.p.) was shown to reduce intake of ethanol, but also of a sweet solution, in Indiana P alcohol-preferring rats (Le et al., 2005). In addition, lofexidine (50, 100 μg/kg, i.p.) was shown to reduce operant alcohol self-administration in outbred Wistar rats (Rasmussen et al., 2014) and repeated guanfacine treatment (300, 600 μg/kg, i.p.) to decrease alcohol intake, alcohol-deprivation effect, and cue/priming-induced reinstatement of alcohol seeking again in outbred Wistar rats (Fredriksson et al., 2015). α2 AR agonists, including clonidine and guanfacine, have also been shown to block stress-induced reinstatement of nicotine, alcohol, heroin, and cocaine seeking and to reduce craving for cocaine and nicotine, while the α2 AR antagonist yohimbine, accordingly, induces reinstatement (Erb et al., 2000; Lee et al., 2004; Le et al., 2005; Platt et al., 2007; Weinshenker and Schroeder, 2007; Zislis et al., 2007; Sinha et al., 2011; Yamada and Bruijnzeel, 2011; Fox et al., 2012a; Perez et al., 2020). Our data, therefore, strengthen the support for the use of guanfacine to safely reduce excessive alcohol drinking.

The mechanism of the antidrinking effect of α2 AR agonists is not entirely understood. NE levels increase in plasma and cerebrospinal fluid in individuals with AUD during alcohol withdrawal (Glue et al., 1989; Muller et al., 1989; Balldin et al., 1992; De Witte et al., 2003). As AUD develops, excessive NE activity in corticolimbic areas is thought to contribute to the emergence of hyperkatifeia, a greater intensity of negative emotional/motivational signs and symptoms during withdrawal, which then promotes further drinking (Koob, 2008, 2021, 2022; Walker et al., 2008; Koob and Vendruscolo, 2025; Varodayan et al., 2025). While moderate NE levels have beneficial effects on attention and executive function, excessive NE levels result in impaired function in humans (Arnsten and Li, 2005; Arnsten, 2006). The detrimental effects of high NE levels on attention and memory are known to be mediated by α1 AR (Arnsten and Goldman-Rakic, 1985a,b; Cai et al., 1993; Birnbaum et al., 1999; Ramos et al., 2005). Similarly, in the context of alcohol, α1 AR antagonism (i.e., prazosin) is able to reduce alcohol drinking, craving, and stress-induced reinstatement of alcohol seeking in rodents and to facilitate abstinence in humans (Walker et al., 2008; Rasmussen et al., 2009; Simpson et al., 2009, 2018; Le et al., 2011; Fox et al., 2012b). On the other hand, α2 AR mediates the effects of moderate NE levels thanks to its high affinity for NE (Wang et al., 2007; Harris et al., 2018). Notably, a downregulation and a reduction in α2 AR function have been reported in humans and animal models (Szmigielski et al., 1977, 1989; Glue et al., 1989; Muller et al., 1989; Balldin et al., 1992; De Witte et al., 2003), and the subsensitivity of α2 ARs has been hypothesized to be a marker of alcohol addiction (Balldin et al., 1992; Berggren et al., 2000). It is, therefore, conceivable that α2 AR agonists may act by normalizing the receptor subsensitivity brought about by chronic alcohol-induced excessive NE release.

The action of α2 AR agonists in reducing physical signs of withdrawal is classically explained by their dampening of the sympathetic nervous system hyperactivity, as NE neurotransmission is enhanced during opioid and alcohol withdrawal (Parale and Kulkarni, 1986; Glue and Nutt, 1987). Specifically, the activation of α2 ARs locally within the LC results in decreased central NE activity and therefore reversal of the sympathetic hyperactivity (Washton and Resnick, 1980; Parale and Kulkarni, 1986). Whether the same mechanism is responsible for the reduction in alcohol intake is unknown, but it is, in our opinion, unlikely. Indeed, it has now been established that the vast majority of α2 ARs is localized postsynaptically to NE terminals (Stenberg, 1989; Arnsten and Cai, 1993; Ramos et al., 2006; Wang et al., 2007; Harris et al., 2018) and a key study has shown that, although α2 ARs expressed on NE elements (i.e., autoreceptors) do cause a decrease in NE neuronal firing and NE release, most behavioral effects of α2 AR agonists are mediated by α2 AR activation on non-NE neurons (i.e., heteroreceptors; Gilsbach et al., 2009; and see also Shaham et al., 2000). Importantly, the extensive and elegant studies from the Arnsten group in nonhuman primates have clearly determined that low doses of guanfacine exert their procognitive effects via postsynaptic α2 heteroreceptors (Arnsten and Li, 2005; Wang et al., 2007). Therefore, we hypothesize that chronic alcohol consumption may induce a long-lasting NE release onto postsynaptic α2 AR heteroreceptors in brain regions such as the PFC and perhaps the extended amygdala and that the potential resulting desensitization of α2 ARs would result in increased ethanol drinking (e.g., as with the removal of a “brake”). The treatment with an α2 AR agonist, such as guanfacine, would, therefore, restore this control.

People affected by AUD and other addictions to substances show profound cognitive impairments, particularly in areas that depend on the PFC; addiction is indeed characterized by poor impulse control, poor attention regulation, impaired working memory and organization, and impaired regulation of emotion (Gould, 2010; Dominguez-Salas et al., 2016; Bruijnen et al., 2019; Melugin et al., 2021; Ceceli et al., 2022; Gasparyan et al., 2023), deficits which resemble those of individuals with damage to the PFC (Rogers et al., 1999). Similar deficits in working memory tasks that depend on the integrity of the mPFC can indeed be reproduced in a variety of animal models of alcohol exposure (Cippitelli et al., 2010; George et al., 2012; Kroener et al., 2012; Gass et al., 2014; Trantham-Davidson et al., 2014; Charlton et al., 2019; Athanason et al., 2023). This PFC dysfunction is hypothesized to sustain compulsive alcohol drinking, trigger relapse, and hinder the success of treatment in AUD (Abernathy et al., 2010; Koob and Volkow, 2016; Pahng et al., 2017). In the PFC, NE has a key role in modulating cognitive function, often by increasing “signals” relative to “noise.” In contrast to what occurs in the posterior cortices and subcortical structures, where NE enhances neural processing through actions at β and α1 ARs, the cognitive processes mediated by NE in the PFC are relatively unaffected by β AR, impaired by α1 AR, especially during stress, and markedly improved by α2 AR activation (Li and Mei, 1994; Tanila et al., 1996; Li et al., 1999; Mao et al., 1999; Arnsten, 2000; Arnsten and Jin, 2014). Guanfacine has the ability to improve PFC connectivity and function and, because of that, is used in ADHD, a disorder characterized by symptoms of PFC dysfunction (Arnsten, 2020; Faraone et al., 2024). α2 AR agonist effects of enhancing signal-to-noise ratio are more evident in conditions of high distraction, which necessitate PFC function for optimal performance (Jackson and Buccafusco, 1991; Wang et al., 2007; Brennan and Arnsten, 2008; Arnsten, 2009). Despite the recognized cognitive-enhancing effects of α2 AR agonism, this has not been established in the context of alcohol. Here, we show that guanfacine is able to improve cognitive performance in mice with a history of chronic alcohol drinking. Notably, guanfacine was particularly effective in a test of temporal order; guanfacine also improved performance, though to a lesser extent, in a test of novel object recognition. The α2 AR agonist, on the other hand, was not effective in a test of novel spatial location. These data appear to suggest that guanfacine acts as a cognitive enhancer following chronic ethanol exposure, in particular when relative recency judgments are involved.

The PFC has a central role in the encoding and the recall of the recency of events in time (or temporal memory); subjects with frontal lobe damage, in fact, perform poorly in temporal order judgements even though their recognition memory is good (Shimamura et al., 1988; Milner et al., 1991; Fuster, 2000). Analogously, animals with mPFC lesions struggle with discriminating between objects or locations based on the order in which they were encountered and also struggle with the sequential execution of actions necessary for certain tasks (e.g., nest building; Wikmark et al., 1973; Kesner and Holbrook, 1987; Afonso et al., 2007). Similarly, the cognitive impairments induced by chronic alcohol consumption have been shown to be more evident in tasks that require temporal order judgement (recency memory) as compared with item recognition, which appear to be either spared or affected to a lesser extent (Meudell et al., 1985; Salmon et al., 1986; Squire, 1987). Therefore, our results appear to suggest that α2 AR, in ethanol drinking mice, may not play a major role in cognitive processes that depend on the hippocampus but is highly implicated in those that depend on the PFC (and likely its connections with the perirhinal cortex; Hannesson et al., 2004) and that α2 AR agonism may normalize some of the executive function deficits caused by AUD by enhancing cognitive control. Further pointing to a PFC NE dysfunction in addiction and to key role of PFC α2 AR, guanfacine-induced attenuation of cocaine craving in cocaine-dependent individuals was shown to be associated with an increase in PFC activity (Fox et al., 2012a) and the systemic administration of guanfacine to normalize alcohol-induced dysregulation of PFC glutamate transmission (Fredriksson et al., 2015). Furthermore, NE depletion in PFC is able to decrease alcohol consumption and preference and to prevent alcohol-induced conditioned place preference (Weinshenker et al., 2000; Ventura et al., 2006). It is conceivable that the PFC may be a site of action of the antidrinking effects of α2 AR agonists. A limitation of this study is that only alcohol drinking mice were included in the cognitive study; therefore, whether performance was impaired under vehicle conditions cannot be determined and future studies will be needed to directly compare the effects of guanfacine in ethanol-experienced versus ethanol-naive mice.

NE is synthesized in specific brainstem nuclei, including the locus ceruleus (LC, A6) and the nucleus tractus solitarius (NTS, A2), among other nuclei; from here, NE is released via the DNB and the VNB, respectively, throughout the brain (Moore and Bloom, 1979; Sawchenko and Swanson, 1981; Robertson et al., 2013). Research in the NE and drug abuse field has so far been LC centric and mostly disregarded other NE nuclei of potential critical importance. Our immunohistochemical data showed that 8 weeks of chronic, intermittent ethanol drinking persistently activates NE neurons not only in the LC, but also in the NTS, as measured by an increase in number of neurons that express the catecholaminergic synthetic enzyme TH as well as the marker of sustained neuronal activation DeltaFosB. This increase was observed when alcohol was no longer onboard, during acute withdrawal (24 h). Therefore, our data suggest that chronic alcohol drinking may cause a persistent increased release of NE synthetized in the LC and NTS in efferent brain regions, which in turn likely plays a role in sustaining excessive drinking and hyperkatifeia.

The LC contains >50% of the total number of NE expressing neurons in the brain and projects to multiple brain areas, including orbitofrontal cortex, anterior cingulate cortex and medial PFC, hippocampus, central and basolateral amygdala, hypothalamus, VTA, and striatum (Foote et al., 1983; Chandler et al., 2014). LC NE neurons are activated by an acute administration of ethanol (Chang et al., 1995; Thiele et al., 1997; Lee et al., 2011), as well as by more prolonged ethanol exposures, such as liquid diet and drinking in the dark procedure (Retson et al., 2015; Burnham and Thiele, 2017). Studies have also shown the involvement of specific NE pathways originating in the LC (e.g., to the VTA, lateral hypothalamus, rostromedial tegmental nucleus, etc.) in ethanol rewarding effects as well as in drinking behavior (Shelkar et al., 2017; Burnham et al., 2021; Dornellas et al., 2021). Different LC NE projections likely enable the differential modulation of diverse types of behaviors and functions. To further complicate the picture, a couple of elegant studies have shown that different LC NE neuronal activation patterns can drive opposing behaviors; indeed, low-frequency tonic firing rates increase alcohol consumption and drive anxiety-like behavior and aversion via α1 AR, while phasic firing decreases alcohol consumption and does not induce aversion (McCall et al., 2015; Deal et al., 2020; perhaps because of α2 AR activation). Interestingly, while tonic responses of LC-NE neurons are elicited by stressful stimuli, phasic responses are produced by salient stimuli (Aston-Jones and Bloom, 1981; Sara and Bouret, 2012), so it is conceivable that different patterns and/or durations of alcohol intake may result in different firing responses as well.

The NTS, which projects to the hypothalamus, parabrachial nucleus, dorsal nucleus of the vagus nerve, LC, CeA, and BNST, among other regions (Delfs et al., 2000; Smith and Aston-Jones, 2008; Mejias-Aponte et al., 2009), is also shown to be activated by acute ethanol (Thiele et al., 2000; Vilpoux et al., 2009; Lee et al., 2011; Aimino et al., 2018; Vazey et al., 2018; Robinson et al., 2020); however, its potential activation by and role in chronic alcohol consumption is less established and reports are sparse (Burnham and Thiele, 2017). Notably, several studies have revealed a role for NE NTS neurons specifically, but not for NE LC neurons, in different drug-related behaviors, such as drug-induced conditioned place preference and aversion, and reinstatement of drug seeking behavior (Shaham et al., 2000; Wang et al., 2001; Olson et al., 2006); furthermore, lesion of NE neurons of the LC do not affect the ameliorating action of clonidine on opiate withdrawal (Britton et al., 1984; Caille et al., 1999). Interestingly, α2 AR agonists are able to suppress footshock-induced release of NE both in the PFC, which is a major terminal region of the NE system arising from the LC (but not from the NTS), and in the amygdala, which receives input from both LC and NTS. The NTS has indeed important projections to the extended amygdala (CeA and BNST), and CeA projecting NTS NE neurons are known to be activated by emotionally salient stimuli (Myers and Rinaman, 2005). Based on these differential projections, the LC→PFC NE pathway would likely be the one mediating the cognitive impairment observed in ethanol drinking mice and likely associated to the guanfacine-induced improvement of performance observed in the cognitive tests (which are heavily PFC dependent). Our finding of sustained activation of NE neurons in the NTS after chronic alcohol drinking warrants a deeper investigation of the role of NE pathways originating in this nucleus in alcohol-related behaviors. Future studies will be needed to probe which exact projection/projections are persistently activated by chronic alcohol consumption and the downstream regions responsible for each of the effects of α2 ARs agonists. In addition, while we assessed TH + DeltaFosB levels in mouse brains that were collected 24 h after the last ethanol drinking session, it will be important in future work to assess activation levels also at longer withdrawal times to determine how long they stay activated during abstinence. Finally, our immunohistochemical experiment was designed as an initial screen to identify ethanol-responsive NE nuclei and did not test whether guanfacine modulates or normalizes this ethanol-induced activation, which may represent an important direction for future work. Due to the widespread projections of LC and NTS NE neurons, further studies are warranted to establish whether NE regulates different aspects of alcohol addiction (e.g., excessive drinking, anxiety-like behavior, cognitive impairment, hyperalgesia, etc.) via different neuronal pathways, with different firing rates, and/or different receptor subtypes.

In animals exposed to chronic, intermittent alcohol drinking, the animal model used in this study, physical (somatic) signs of withdrawal are generally very mild or absent (Hwa et al., 2011); on the other hand, affective signs of withdrawal and hyperkatifeia are commonly reported and include negative affect-like behavior and increased sensitivity to pain (Quadir et al., 2020, 2021a,b; Centanni et al., 2022; Peng et al., 2024). Hence, we can conclude that the antidrinking effect of α2 AR agonists is likely independent from any actions of physical signs, while an effect on anxiety-like behavior and other negative affective states may have been present, based on literature showing that α2 AR activation has anxiolytic-like effects (Morrow et al., 2004; Langen and Dost, 2011; Buffalari et al., 2012; Fox et al., 2015).

While most of the measures of the effect of clonidine and guanfacine in this series of study were found to be comparable between sexes, guanfacine appeared to be more potent in improving temporal order memory in female mice, as compared with males. This may have been due to the lower baseline of females in this test under vehicle conditions, or it may reflect that females may respond more to the beneficial effects of guanfacine, as shown before, especially in stressful and emotionally challenging test conditions (Fox et al., 2014; Milivojevic et al., 2017; but also see Shansky et al., 2009; Mineur et al., 2015). A limitation of this study is that the LORR and body temperature assays were conducted only in male mice; future studies including females in these sedative effect measures will help strengthen and generalize the findings.

Taken together, our data support the hypothesis that α2 AR agonism represents a highly promising therapeutic target to reduce alcohol consumption and associated cognitive deficits in AUD.

## Synthesis

Reviewing Editor: Leandro Vendruscolo, National Institute on Drug Abuse Decisions are customarily a result of the Reviewing Editor and the peer reviewers coming together and discussing their recommendations until a consensus is reached. When revisions are invited, a fact-based synthesis statement explaining their decision and outlining what is needed to prepare a revision will be listed below. The following reviewer(s) agreed to reveal their identity: Sebastien Carnicella.

Reviewer 1

The authors totally avoid this part of my comments, which was critical: "However, the study sounds incomplete at this stage to clearly answer the two objectives of the study (less/lack of undesirable and how/why guanfacine reduces alcohol intake). The questions raised below will be important to answer in order to improve the value of the present study in regard to the existing literature regarding guanfacine and AUD."

As they did not answer at all to this concern (they decided to not provide more data), the study remains too incomplete to be suitable for publication.

Reviewer 2

This manuscript addresses clinically relevant treatments for alcohol use disorder with valuable findings. However, it needs major revisions to be adequately reviewed for publication:

Abstract

1) The instructions for authors for this journal state that abstracts are limited to 250 words. Please revise.

Introduction

2) The instructions for authors for this journal state that introductions are limited to 750 words including citations. Please revise.

3) For the section starting with, "Guanfacine has also been shown to reduce craving for cocaine and nicotine.." (second paragraph of introduction), specify whether these findings were in humans or rodents.

Methods

4) 2.6: Throughout each subsection (TOM, NOR, NSL) there are explanations of how each behavior indicates aspects of memory retrieval. The authors should provide citations for these statements.

5) 2.7: The authors should state at what time point brains were collected for IHC and how long after a behavioral test. Authors should also specify if this is a new cohort of mice.

6) 2.7: Small typo at the end of the Quantification section: "One hemisphere was randomly chosen for each section; the contours of the areas of interest were drawn at using an Olympus..."

7) 2.8: The authors should state how body temperature data was analyzed and what post hoc analyses were used.

8) 2.8: The statistical analyses report using "student's Dunnet's and Duncan's tests". "Student's" should be removed for accuracy. Further, it is reported that Student's t-tests were used as post-hoc comparisons, but t-tests do not have corrections for multiple comparisons. Authors should use appropriate post-hoc analyses with corrections for multiple comparisons or justify the use without. Which tests were used could also be clarified throughout the results as multiple methods were used for post-hoc analysis.

9) The authors should add specifics for all chemicals, reagents, drugs, and apparatuses throughout the methods section (e.g., v/v, location/company purchased from) and in accordance with the instructions for authors, "All companies from which materials were obtained should be listed. If materials were obtained from an individual, an affiliation for that individual should be listed."

Results

10) P-values can be limited to 3 or 4 digits.

11) 3.1: Why was a one-way ANOVA conducted instead of post hoc analyses within the two-way ANOVA? This should be clarified as this type of analysis would not correct for multiple comparisons and could artificially inflate results. Additionally, the authors state, "Indeed, clonidine administration significantly reduced body temperature at all time points tested, according to one-way ANOVAs, except at 240 min.", however, Figure 1A does not show significance at 120 min, implying that a significant reduction was not observed at 120 or 240 min.

12) 3.1: The beginning of the third paragraph states, "Even though the no ethanol and ethanol tests were run at different times, and so no direct statistical comparisons can be made between the two, the basal temperature levels did not differ between the two groups..." However, in the first paragraph of this section, the authors ran and reported a direct statistical comparison between ethanol and no-ethanol groups using a three-way ANOVA. If they were run at different times, they should not be compared statistically but should be commented on. If I am mistaken of my understanding of these analyses, please clarify in that section further for readability.

13) 3.1: In the fourth paragraph, authors should specify what groups were collapsed for the post-hoc comparisons. It is mentioned that sex was collapsed in the statistical methods, but this should only occur if interactions with sex are not reported. Main effects of Sex and Time are missing in many of the results reported.

14) 3.3: The authors state, "Post-hoc analysis showed that all three doses of guanfacine (12.5, 25, and 50 ug/kg) significantly reduced ethanol intake at both time points..." however, the post-hoc analyses presented in figure 3B show that 12.5 guanfacine did not reduce ethanol intake in the 6 h assessment. This should also be adjusted in the discussion where it states that all the doses tested were effective.

15) 3.5: In the TOM test section, that authors state that data were pooled together, however, an interaction was observed. Therefore, statistics should not be run with sexes together and the data should only be presented to explain the interaction. Further, the discussion should reflect this.

16) 3.6: Statistics for Figure 6B report p = 0.059, but the figure indicates p < 0.05 as a "*".

Figures

17) In the extended data 2.1 Aand B: are post hoc analyses missing from these graphs?

18) Just double checking that there is not a statistical outlier in Figure 3C for Guanfacine 25 ug/kg.

Discussion

19) In accordance with eNeuro article guidelines, "If animals, particularly rodents, were housed individually in single cages, the possible caveats for data interpretation should be discussed." The authors should add a statement to this effect to the discussion.

20) The authors state they have streamlined the Discussion; however, it is still currently 3,861 words when the limit as stated on the "Information for Authors" instructions is 3,000 words including citations.

Overall

21) Spell out all abbreviations the first time it is used before abbreviating throughout (e.g., intraperitoneal, immunohistochemistry, etc.)

22) Authors should proofread throughout.

23) Clonidine and guanfacine are generic drug names and should not be capitalized except when beginning a sentence.
