# Supplementary figures and images for: Alpha-2 Adrenergic Agonists Reduce Heavy Alcohol Drinking and Improve Cognitive Performance in Mice
Source: eNeuro. 2026 Jan 29;13(1):ENEURO.0368-25.2026. doi: 10.1523/ENEURO.0368-25.2026 (PMC12866760; doi:10.1523/ENEURO.0368-25.2026)

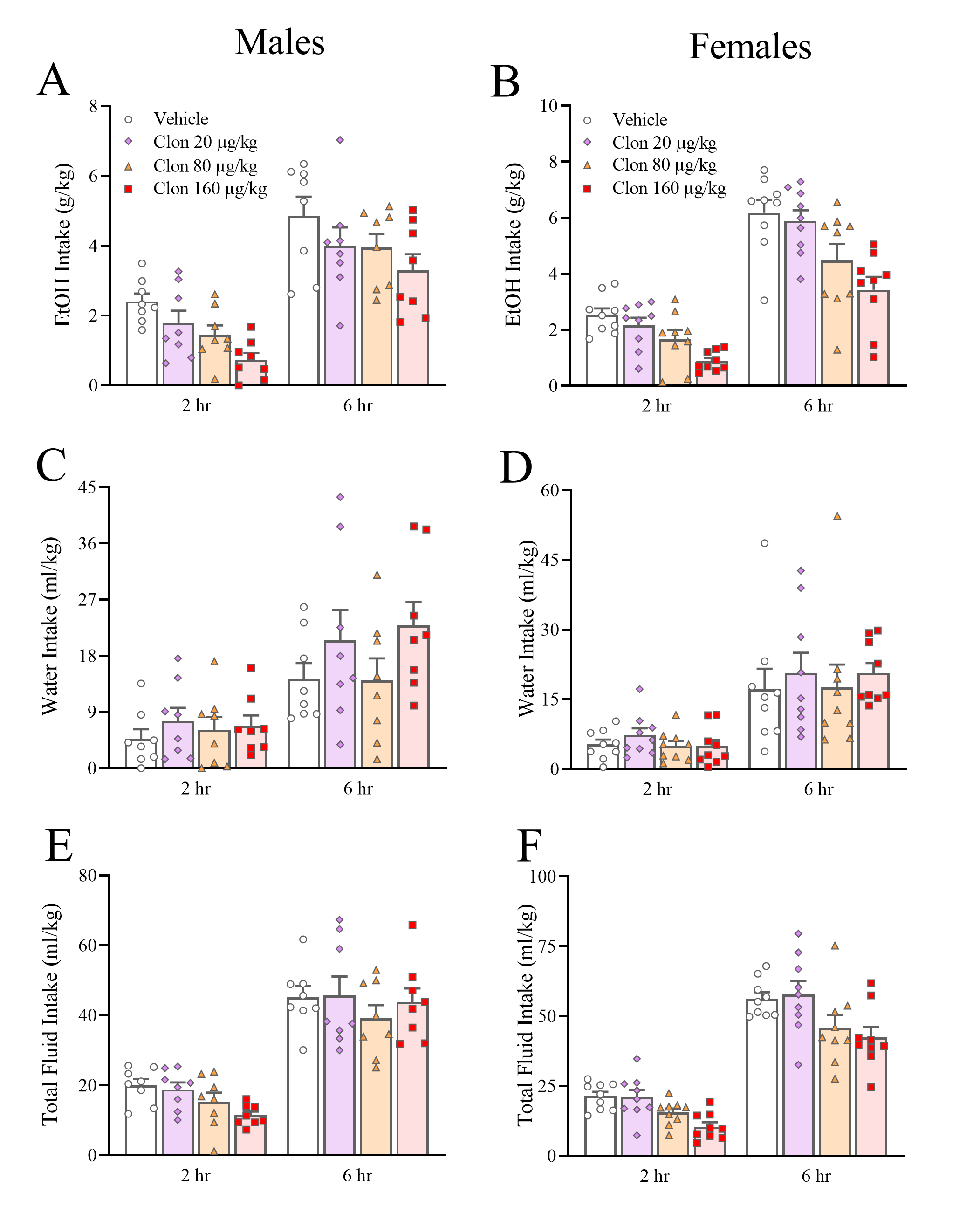

Supplement: Figure 2-1 — Male and female C57Bl/6J mice exposed to intermittent access to alcohol (IA2BC) were administered Clonidine (Clon, 0-160 µg/kg, i.p.). Data are here reported disaggregated by sex: (A, C, E) males, (B, D, F) females. (A, B) 2 h and 6 h ethanol intake, (C, D) 2 h and 6 h water intake, (E, F) 2 h and 6 h total fluid intake. Data represent Mean ± SEM (in each sex, n = 8-9/group). Download Figure 2-1, TIF file. [file eneuro-13-ENEURO.0368-25.2026-s002.tif]

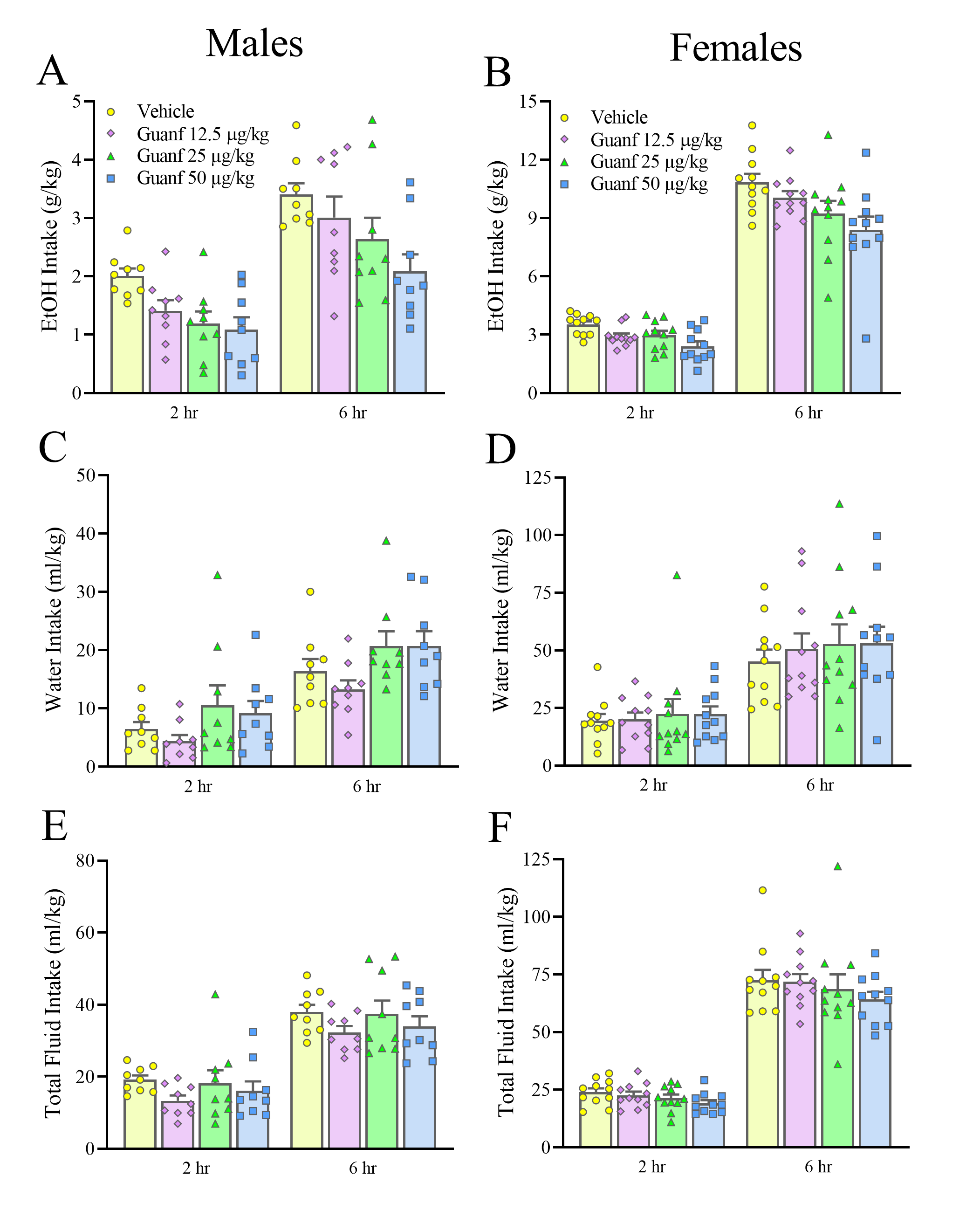

Supplement: Figure 3-1 — Male and female C57Bl/6J mice exposed to intermittent access to alcohol (IA2BC) were administered Guanfacine (Guan, 0-50 µg/kg, i.p.). Data are here reported disaggregated by sex: (A, C, E) males, (B, D, F) females. (A, B) 2 h and 6 h ethanol intake, (C, D) 2 h and 6 h water intake, (E, F) 2 h and 6 h total fluid intake. Data represent Mean ± SEM (in each sex, n = 8-9/group). Download Figure 3-1, TIF file. [file eneuro-13-ENEURO.0368-25.2026-s003.tif]
